# Susceptibility of different mice species to chemical induction of colorectal cancer by 1,2-dimethylhydrazine

**DOI:** 10.1186/s43046-024-00255-x

**Published:** 2025-01-13

**Authors:** Nourhan M. Abdelmaksoud, Ahmed I. Abulsoud, Tamer M. Abdelghany, Shereen Saeid Elshaer, Ahmed Samaha, Nadine W. Maurice, Sherine Maher Rizk, Mahmoud A. Senousy

**Affiliations:** 1 Department of Biochemistry, Faculty of Pharmacy, Heliopolis University, 3 Cairo-Belbeis Desert Road, P.O. Box 3020El Salam,, 11785 Cairo, Egypt; 2 Department of Biochemistry and Molecular Biology, Faculty of Pharmacy (Boys), Al-Azhar University, Nasr City, Cairo, 11823 Egypt; 3 Department of Pharmacology and Toxicology, Faculty of Pharmacy (Boys), Al-Azhar University, Nasr City, Cairo, 11884 Egypt; 4 Department of Pharmacology and Toxicology, Faculty of Pharmacy, Heliopolis University, 3 Cairo-Belbeis Desert Road, P.O. Box 3020El Salam, 11785 Cairo, Egypt; 5 Department of Biochemistry and Molecular Biology, Faculty of Pharmacy (Girls), Al-Azhar University, Nasr City, Cairo, 11823 Egypt; 6 Department of Pharmaceutical and Pharmacological Sciences, University of Padova, 35131 Padua, Italy; 7 Department of Biochemistry, Faculty of Pharmacy, Cairo University, Cairo, 11562 Egypt; 8 Department of Biochemistry, Faculty of Pharmacy and Drug Technology, Egyptian Chinese University, Cairo, 11786 Egypt; 9 Integrative Health Center, Faculty of Pharmacy, Heliopolis University, Cairo, Egypt

**Keywords:** Colorectal cancer, Mouse species, Dimethylhydrazine, Animal model

## Abstract

**Background:**

Colorectal cancer (CRC) is a major public health concern. Animal models play a crucial role in understanding the disease pathology and development of effective treatment strategies. Chemically induced CRC represents a cornerstone in animal model development; however, due to the presence of different animal species with different genetic backgrounds, it becomes mandatory to study the susceptibility of different mice species to CRC induction by different chemical entities such as 1,2-dimethylhydrazine (DMH). This study aimed to investigate the induction receptivity of two commonly used mice species, C57BL/6 and BALB/c, to DMH-induced CRC.

**Methods:**

Both mice species were exposed to weekly intraperitoneal injections of DMH at a dose of 20 mg/kg body weight for 15 consecutive weeks. The response to DMH was evaluated by monitoring body weight gain, daily food intake, and gastrointestinal symptoms. At the end of exposure, histopathology of distal colon dissected from both species was analyzed.

**Results:**

Results revealed that C57BL/6 had a higher response to DMH compared to BALB/c. A significant decrease in body weight gain concomitant with severe diarrhea was observed in C57BL/6 receiving DMH compared to their controls, without any difference in food intake. Histopathology of distal colon revealed aberrant crypt foci and loss of goblet cells in DMH-exposed C57BL/6 mice. On the other hand, BALB/c mice displayed a normal and intact colon, with a normal weight gain pattern, and without any gastrointestinal symptoms.

**Conclusion:**

In conclusion, C57BL/6 has a higher susceptibility toward chemical induction to CRC; therefore, it can be used to study CRC pathogenesis, prevention, and treatment.

## Introduction

Colorectal cancer (CRC) is considered the third cause of cancer-related mortality in both sexes with a higher percentage in males worldwide [1]. Westernization, family history, high body mass index, and consumption of high saturated fat, red meat, and low vegetables are considered the most common risk factors for CRC [2]. In addition, chronic cellular damage caused by increased reactive oxygen species production, oxidative stress-induced DNA damage, and genetic mutations are among the metabolic changes that precede precancerous cell development [3].

Animal models could shed light on the underlying mechanisms involved in CRC development [4]. The commonly used chemical carcinogen for CRC induction includes 1,2-dimethylhydrazine (DMH), an alkylating procarcinogen that is metabolically converted to its active metabolite, azoxymethane (AOM), in the liver. DMH could be given by different routes of administration, including subcutaneous, intraperitoneal, oral, and intrarectal [5, 6]. A plethora of animal models for CRC have been established since the development of carcinogen-induced rodent models for CRC. Sprague Dawley or Wistar rats are a common model, which results in stable CRC-induced models [7–9], while CRC mice models include different species such as BALB/c and C57BL/6 [10–12]. However, there is controversy in the literature about the dose, exposure protocols, and mice species. For instance, weekly intraperitoneal (i.p.) injections of DMH for 6 weeks at a dose of 20 mg/kg has been found to induce CRC in BALB/c mice [13]. In another study, weekly i.p. injections of DMH for 12 weeks at a dose of 20 mg/kg was required for induction of CRC [12]. The same dose was used for CRC induction; however, it was injected subcutaneously twice a week for 24 weeks [14]. On the other hand, i.p. injection of DMH at a dose of 20 mg/kg/week for 8 weeks was sufficient to induce CRC in C57BL/6 mice [15]. Another study reported that with the same dosage regimen, CRC is initiated after 4 weeks and completely induced after 32 weeks [16]. While C57BL/6 develops no neoplasms in colon after administration of the same dose for 15 weeks [17], another study reported that the i.p. administration of 40 mg/kg once weekly for 5 weeks resulted in aberrant crypt foci in the same species [18].

This discrepancy in the doses required, time needed, and exposure protocol hampered the ability to establish a reliable model for studying CRC induction, pathogenesis, prevention, and treatment. Accordingly, this study seeks to establish a chemically induced CRC animal model using DMH and pinpoint the most vulnerable mouse species in Egypt to pave the way for developing more targeted and effective CRC treatment strategies.

## Methodology

### Animals

Twelve BALB/c and 12 C57BL/6 mice, 6 weeks old, male, with an average weight of 25 g, were purchased from Theodor Bilharz’s animal house. Animals were housed in plastic cages (30 × 20 × 13 cm) under controlled environmental conditions, six per cage, in a chemical-free room, artificially illuminated (12-h dark/12 light cycle), and thermally controlled (25 ± 2 °C), with 55 ± 10% humidity at the Animal Facility, Faculty of Pharmacy, Heliopolis University. A standard chow diet and free access to water were provided ad libitum.

### Ethical statement

The experiment protocol was approved by the research ethics committee for experimental and clinical studies of the Faculty of Pharmacy, Cairo University (approval # BC3000).

### Chemicals

1,2-dimethylhydrazine was provided by Sigma-Aldrich, St. Louis, MO, USA, with CAS number: 306–37-6.

### Biochemical assay

Serum was separated, after collecting blood, within 30 min by centrifugation of completely coagulated samples at 2000 × *g* for 10 min at room temperature. Albumin was assayed using an Albumin enzyme-linked immunosorbent assay (ELISA) kit, Cat.No. E-EL-M3032, purchased from E-lab Sciences, USA. Total protein was assayed using Bradford protein colorimetric assay kit, Cat.No. E-BC-K168-M, E-lab Sciences, USA.

### Induction protocol

After 1 week of acclimatization to the laboratory environment, each mouse species was divided into two groups; group I, received a weekly dose of 0.9% normal saline, 10 ml/kg, i.p. for 15 weeks representing the control group, and group II, received a weekly dose of DMH in 0.9% normal saline, 20 mg/kg, i.p. for 15 weeks representing the induction group. DMH was prepared immediately before use in 0.9% saline and was injected i.p. at 20 mg/kg for 15 weeks [12]. Body weight was observed at the initiation and end of the experiment.

The animals were anesthetized with 3% isoflurane and euthanized via cervical dislocation at the end of the experiment. Blood samples were collected by cardiac puncture. Hepatic and colonic tissue samples were dissected and fixed in 10% neutral buffered formalin for 72 h. Samples were processed in serial grades of ethanol, cleared in xylene, and then embedded in paraplast tissue embedding media. Four-micrometer-thick tissue sections were cut by rotatory microtome and mounted on glass slides. Tissue sections were stained with hematoxylin and eosin as a general histological examination staining method, and then examined by an experienced histologist in a blinded manner using a Full HD microscopic imaging system (Leica Microsystems GmbH, Germany) [19].

### Statistical analysis

Statistical analysis was performed using GraphPad Prism, Inc., La Jolla, CA, USA. The statistical differences in both species (control vs DMH-treated group) were calculated using the *t*-test.

## Results

Body weight was observed, and percentage weight gain was calculated according to the following equation: % weight gain = (final weight − initial weight)/final weight x 100.

**Fig. 1:**
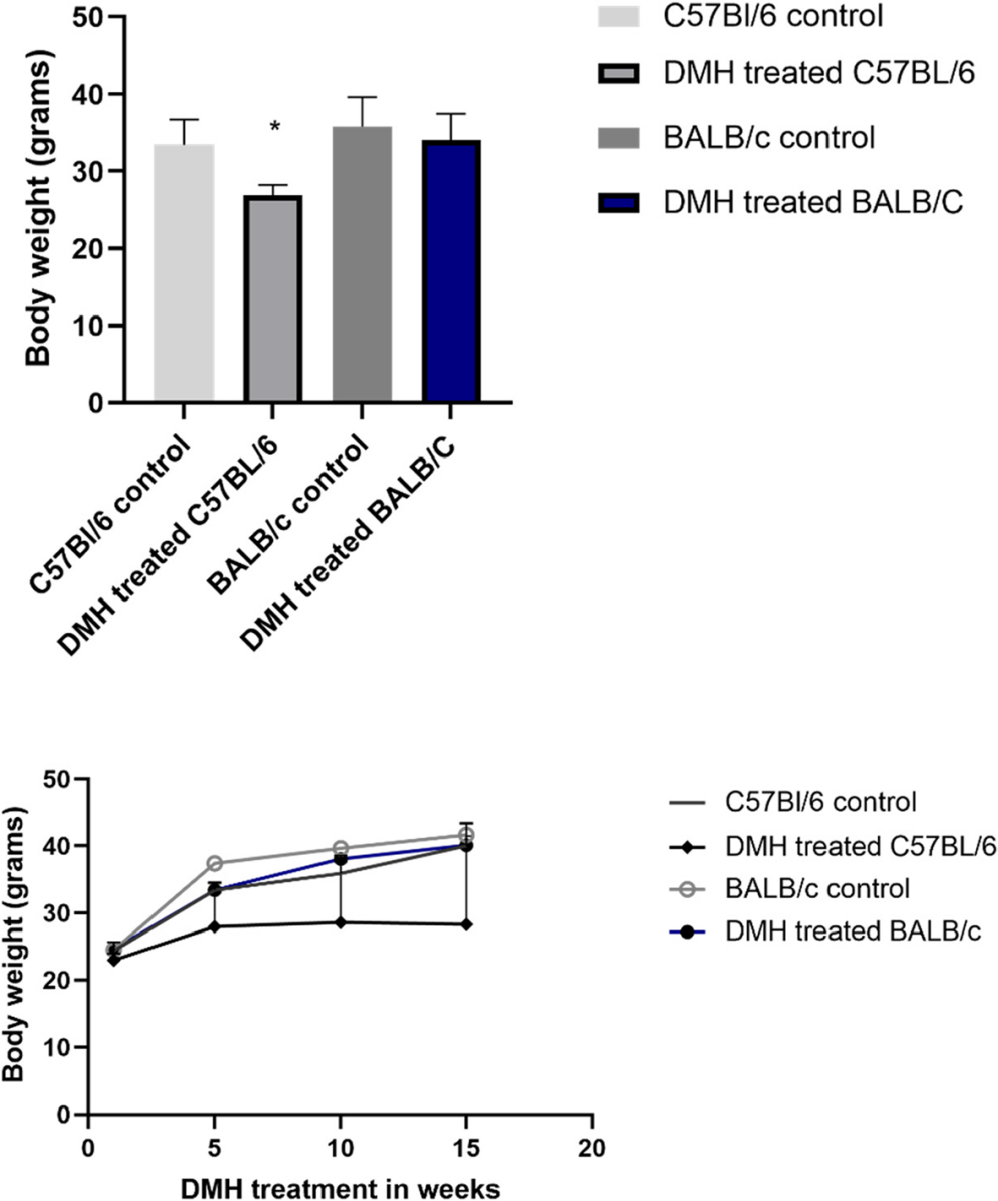
Average body weight (grams) following DMH treatment in BALB/c and C57BL/6 mice. *Statistically significant compared to the control C57BL/6 group. Data are represented as mean ± standard deviation (SD)

**Fig. 2:**
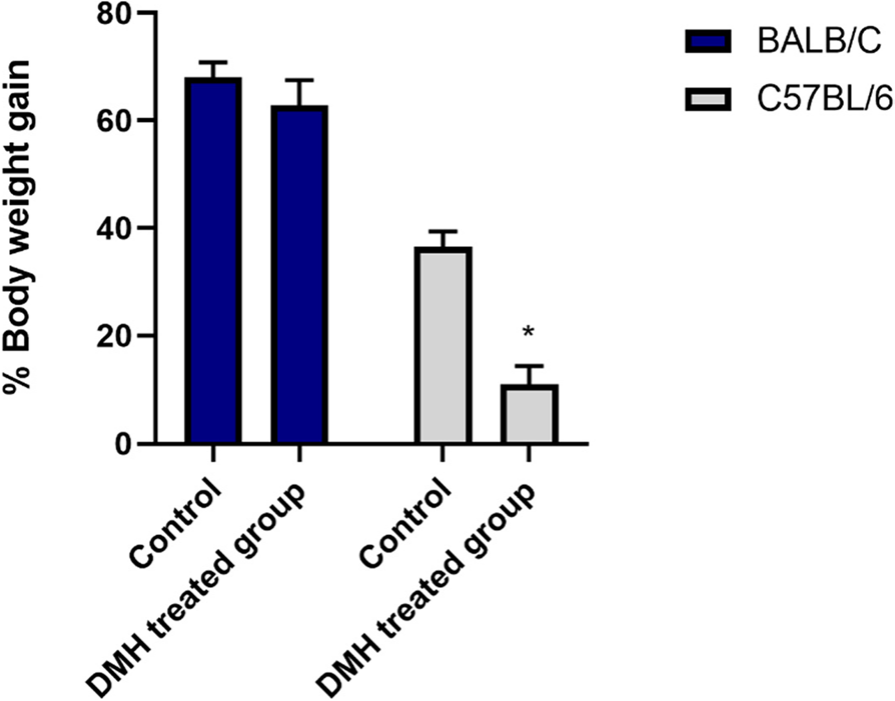
Percent weight gain in BALB/c and C57BL/6 mice. *Statistically significant compared to the control C57BL/6 group. Data are represented as mean percentage ± SD

The initial weight of both species was around 25 g; the average weight in both groups per week of DMH treatment is shown in Fig. 1. The average % weight gain was 68% and 62% in the control and DMH-treated BALB/c mice, respectively, while it was 36% and 11% in the control and DMH-treated C57BL/6 mice, respectively, as shown in Fig. 2. There was a statistically significant decrease in the percent body weight between DMH-treated C57Bl/6 species using *t*-test with *p* value = 0.0092. Moreover, a statistically significant difference of average body weight = 0.025 between the control and DMH-treated C57Bl/6 mice. In addition, C57BL/6 mice experienced diarrhea that was not experienced by BALB/c mice. There was not any noted difference in food intake in either species.

### Histopathological investigation

**Fig. 3:**
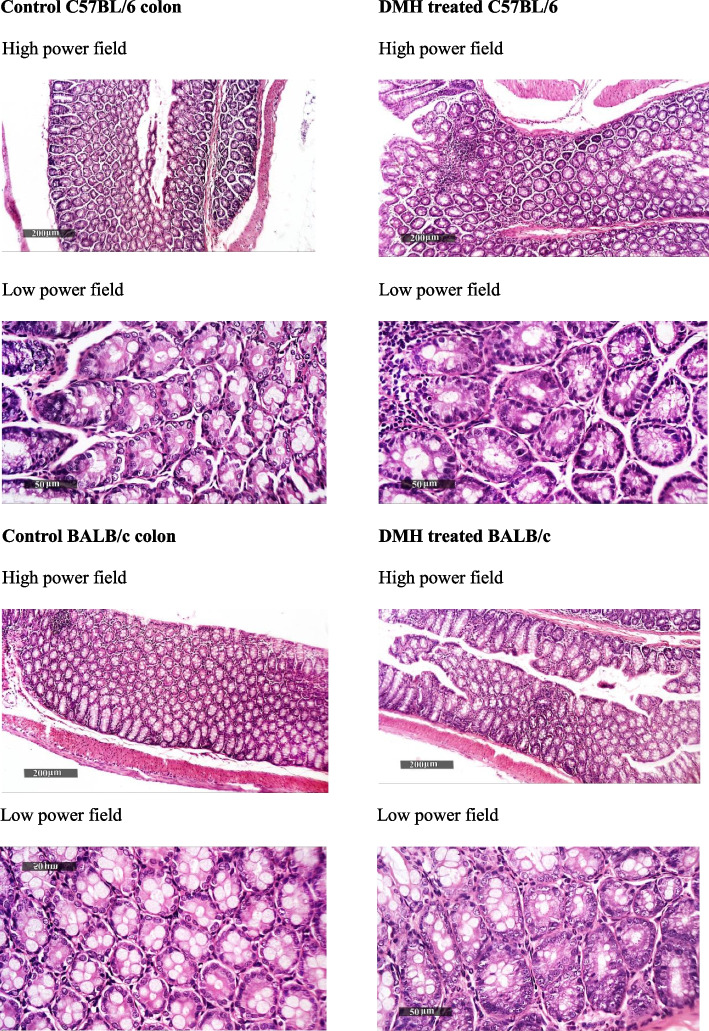
Histopathological examination of control and DMH-treated colon of C57BL/6 and BALB/c in low- and high-power fields

Histopathological examination by hematoxylin and eosin showed a precancerous lesion in the colon of C57BL/6 while there were no lesions in the BALB/c colon. Normal control samples of C57BL/6 mice demonstrated normal organized morphological features of the colon wall with intact colonic crypts showing abundant goblet cells (black star) and intact covering epithelium with normal submucosa (blue star) and outer muscular coat as shown in Fig. 3, while microscopical examination of DMH-treated mice was also revealed in Fig. 3. Compared to control group, C57BL/6 colon showed shrinkage, focal scattered colonic crypts with mild anaplastic changes including hyperchromatic nuclei with moderate overcrowdings and goblet cell loss (red star), adjacent to apparent intact intestinal crypts (black star), accompanied by moderate interstitial lymphocytic and macrophages infiltrates (arrowhead). On the other hand, treated BALB/c colon showed normal organized histological features of colonic crypts resembling normal controls.

### Biochemical analysis

**Fig. 4:**
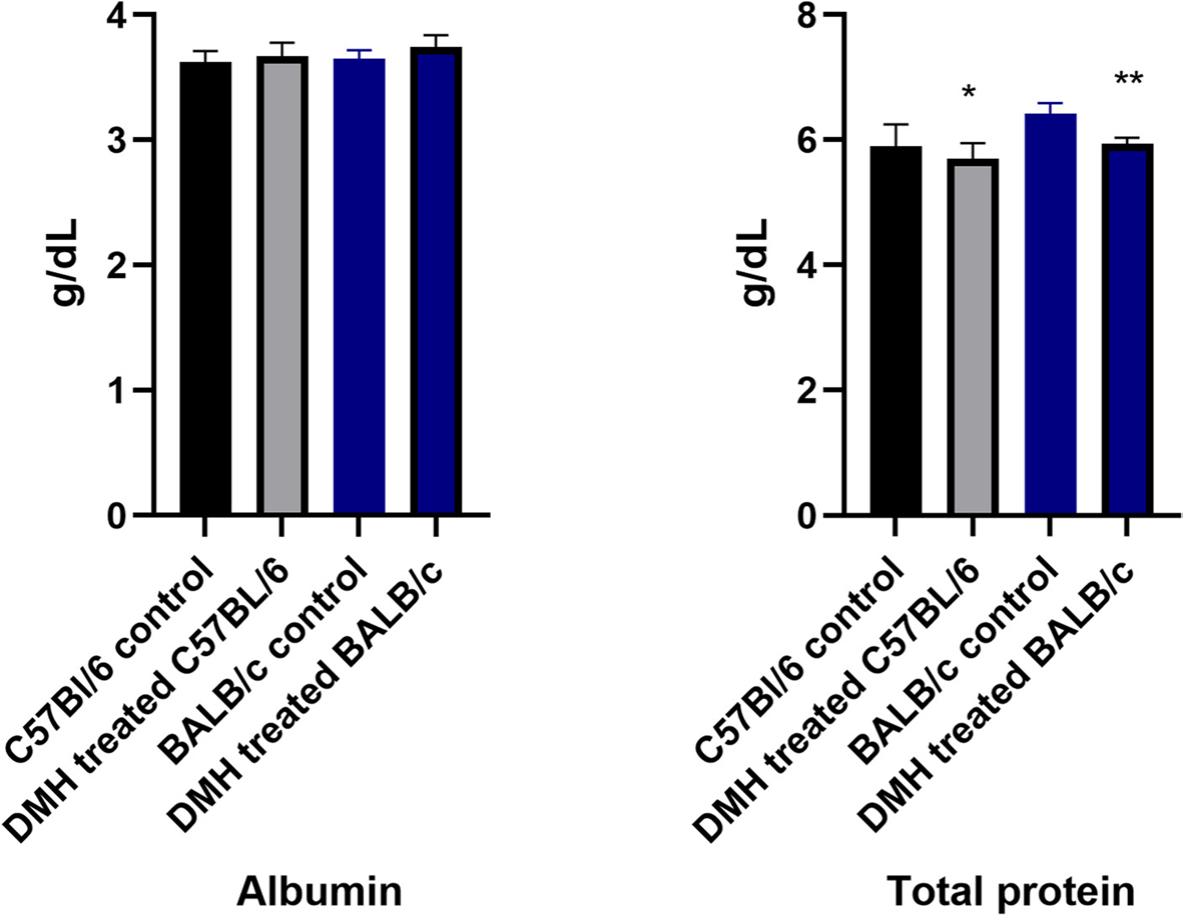
Serum albumin and total protein in control and DMH-treated groups in both species. *Statistically significant compared to C57BL/6 control group *p* < 0.001. **Statistically significant compared to BALB/c control group *p* < 0.001. Data are expressed as mean ± SD

Serum albumin and total protein levels were assayed in both species (Fig. 4). There was a statistically non-significant difference in albumin serum of DMH-treated mice compared to control. However, there were statistically significant differences in serum total protein of DMH-treated groups compared to their control groups.

## Discussion

The analysis of changing patterns in cancer development is important in the development of anticancer drugs. Various species of mice are utilized in CRC research, including inbred and genetically modified species [20, 21]. The choice of mouse model depends on specific research objectives; however, a chemical-induced carcinogen is widely used in animal models utilizing DMH or its active metabolite, AOM, in CRC-induced models [13, 22]. DMH is a pro-carcinogen of the colon that causes DNA methylation, mismatch, and mutation after going through a series of activation reactions in the liver producing AOM and methyl-azoxy methanol [23, 24]. Notably, DMH induces histopathological and molecular changes similar to human CRC [25]. As a result, it is widely used in the laboratory to induce CRC.

The importance of understanding the susceptibility of mice to CRC induction by DMH cannot be overstated. This study delves into this matter by investigating two commonly used mouse species, providing valuable insights that can potentially aid in the development of effective preventative measures against CRC.

Both mice species were i.p. injected weekly with 20 mg/kg body weight of DMH for 15 weeks. The response to DMH was observed by reduced weight gain without a change in diet intake. It may be linked to the associated diarrhea as weight loss and diarrhea are considered CRC signs [26]. Previous results reported decreased weight gain or weight loss in DMH-induced CRC models [27, 28]. On the contrary, another study showed the effect of an anticarcinogen on DMH-treated rats and reported no weight change [29].

Histopathological examination showed aberrant crypt foci, loss of goblet cells, and macrophage infiltrates in the colon of C57BL/6 mice while a normal intact organized colon was found in BALB/c mice. It provides information regarding the susceptibility of C57BL/6 over BALB/c mice to DMH-induced CRC. Severe inflammation and aberrant crypt foci are the histopathological signs of DMH-induced CRC [30, 31]. A previous study investigated the impact of selenium on DMH-induced CRC in BALB/c female mice and demonstrated that the DMH-induced group was associated with aberrant foci; however, the route of administration was subcutaneous injection for 8 weeks [11]. Male C57BL/6 mice showed aberrant crypt foci after 6-week injections of 40 mg/kg DMH in another study [18]. Additionally, BALB/c mice were given subcutaneous injections of DMH with a dose of 20 mg/kg twice a week for a duration of 24 weeks [14]. CRC was induced in male Laca mice by administering subcutaneous injections of DMH 30 mg/kg once a week for 20 weeks [32].

Serum albumin and total protein levels were assayed in both species. Non-significant albumin levels were found in both DMH-treated groups compared to their controls. However, there was a significant decrease in the total protein levels in the DMH-treated groups in both species relative to their corresponding controls, suggesting that DMH did affect kidneys. It comes in agreement with a previous study that reported DNA damage in the kidney following the DMH treatment in different species of mice [33], while it does not affect serum albumin levels in accordance with a previously reported finding that utilized DMH in CRC induction in Wistar rats [34].

The difference in susceptibility between C57BL/6 mice and BALB/c mice to CRC may be due to the difference in cytokines and immune cells involved in their immune response which are categorized into T helper cells (Th-1 and Th-2). Specifically, C57BL/6 mice have overexpressed Th-1 cells while BALB/c mice have Th-2 cells [35, 36]. A recent study discovered that the Th-2 cells in BALB/c mice can resist inflammatory responses associated with colitis by modulating the host’s inflammatory, metabolic, and gut microbial profiles. In contrast, C57BL/6 mice with Th-1 cells were found to experience severe inflammatory responses [37]. Th-1 cells are promoted by interferon-gamma released in C57BL/6 mice more than BALB/c mice [38]; consequently, inflammation is driven by interleukin-12 [39]. These findings support the results of the study and suggest that C57BL/6 mice are more susceptible to CRC than BALB/c mice.

Further studies are recommended using increased doses of DMH or longer duration of treatments for CRC-induced models aiming to construct an established preclinical model for further investigation of CRC pathogenesis and treatments.

## Conclusion

In conclusion, the study explored the susceptibility of different mice species to CRC induced by DMH and its implications for cancer research and therapeutics. The study found that C57BL/6 mice were more susceptible to DMH-induced CRC than BALB/c mice which may be due to genetic variation. Consequently, the findings could be translated into human studies and clinical trials. However, it is essential to consider species-specific differences and limitations. Mice models can aid in the preclinical evaluation of novel therapeutic agents, including chemo-preventive compounds, immunotherapies, and targeted drugs. Additionally, these models can help in assessing the efficacy and safety of potential treatment strategies before progressing to human trials.

## Data Availability

All data generated or analyzed during this study are included in this published article.

## Abbreviations

DMH: 1,2-Dimethylhydrazine
AOM: Azoxymethane
CRC: Colorectal cancer
ELISA: Enzyme-linked immunosorbent assay
i.p.: Intraperitoneal
Th: T helper cell
